# Technology-supported sitting balance therapy versus usual care in the chronic stage after stroke: a pilot randomized controlled trial

**DOI:** 10.1186/s12984-021-00910-7

**Published:** 2021-07-28

**Authors:** Liselot Thijs, Eline Voets, Evelien Wiskerke, Thomas Nauwelaerts, Yves Arys, Harold Haspeslagh, Jan Kool, Patrick Bischof, Christoph Bauer, Robin Lemmens, Daniel Baumgartner, Geert Verheyden

**Affiliations:** 1 Department of Rehabilitation Sciences, KU Leuven - University of Leuven, Leuven, Belgium; 2 NiniX Technologies, Bruges, Belgium; 3 Department of Physiotherapy, Kliniken Valens, Valens, Switzerland; 4 IMES Institute of Mechanical Systems, ZHAW School of Engineering, Winterthur, Switzerland; 5 Institute of Physiotherapy, ZHAW School of Health Professions, Forschungsschwerpunkt Biomechanical Engineering, Technikumstrasse 9, 8400 Winterthur, Switzerland; 6 Center for Brain & Disease Research, Laboratory of Neurobiology, VIB, Leuven, Belgium; 7 Department of Neurology, University Hospitals Leuven, Leuven, Belgium; 8 Department of Neurosciences, Experimental Neurology, and Leuven Brain Institute (LBI), KU Leuven – University of Leuven, Leuven, Belgium

**Keywords:** Stroke, Technology-supported, Sitting Balance, Trunk Rehabilitation, Randomized Controlled Trial, Feasibility

## Abstract

**Background:**

Technology development for sitting balance therapy and trunk rehabilitation is scarce. Hence, intensive one-to-one therapist-patient training is still required. We have developed a novel rehabilitation prototype, specifically aimed at providing sitting balance therapy. We investigated whether technology-supported sitting balance training was feasible and safe in chronic stroke patients and we determined whether clinical outcomes improved after a four-week programme, compared with usual care.

**Methods:**

In this parallel-group, assessor-blinded, randomized controlled pilot trial, we divided first-event chronic stroke participants into two groups. The experimental group received usual care plus additional therapy supported by rehabilitation technology, consisting of 12 sessions of 50 min of therapy over four weeks. The control group received usual care only. We assessed all participants twice pre-intervention and once post-intervention. Feasibility and safety were descriptively analysed. Between-group analysis evaluated the pre-to-post differences in changes in motor and functional outcomes.

**Results:**

In total, 30 participants were recruited and 29 completed the trial (experimental group: n = 14; control group: n = 15). There were no between-group differences at baseline. Therapy was evaluated as feasible by participants and therapist. There were no serious adverse events during sitting balance therapy. Changes in clinical outcomes from pre- to post-intervention demonstrated increases in the experimental than in the control group for: sitting balance and trunk function, evaluated by the Trunk Impairment Scale (mean points score (SD) 7.07 (1.69) versus 0.33 (2.35); *p* < 0.000); maximum gait speed, assessed with the 10 Metre Walk Test (mean gait speed 0.16 (0.16) m/s versus 0.06 (0.06) m/s; *p* = 0.003); and functional balance, measured using the Berg balance scale (median points score (IQR) 4.5 (5) versus 0 (4); *p* = 0.014).

**Conclusions:**

Technology-supported sitting balance training in persons with chronic stroke is feasible and safe. A four-week, 12-session programme on top of usual care suggests beneficial effects for trunk function, maximum gait speed and functional balance.

*Trial Registration:* *ClinicalTrials.gov identifier:* NCT04467554, https://clinicaltrials.gov/ct2/show/NCT04467554, date of Registration: 13 July 2020.

**Supplementary Information:**

The online version contains supplementary material available at 10.1186/s12984-021-00910-7.

## Background

Stroke is an important cause of increasing disability-adjusted life years [1], and requires rehabilitation. Rehabilitation after stroke is an intensive process with a multidisciplinary approach aiming to provide optimal independence in activities of daily living (ADL) and to improve social interaction [2]. Motor therapy is an important component of rehabilitation. The focus of motor therapy is often the recovery of arm-hand function and of gait but published research convincingly demonstrates that sitting balance is an important predictor of recovery of ADL. Even in the chronic phase after a stroke, there is a persistent deficit in sitting balance, as well as impaired trunk function [3, 4]. Furthermore, research indicates that by intensifying therapy, as in more therapy time with greater numbers of repetitions, ADL can be improved [5].

Improving sitting balance and trunk function are essential components of rehabilitation after stroke. Several studies have focused on training trunk function, with participants trained, not only in the acute or subacute phase [6–9], but also in the chronic phase [10–22]. On average, the study population in the latter phase received 168 min of trunk therapy per week for 2 to 12 weeks. However, therapy protocols offered in these trials were labour-intensive due to the need for one-to-one interaction between therapist and patient. In addition, research suggests that structured implementation of motor learning principles [23] would be beneficial for improving sitting balance and trunk function. Technology can facilitate this process by delivering an efficient method of offering intensive therapy, reducing the need for continuous input and control by a therapist. This could lead to an increased quantity of therapy of greater intensity. We have therefore developed a novel rehabilitation technology therapy concept, with sitting balance therapy offered on a newly developed device called T-Chair. This development started with an unstable office chair that allowed small, non-automated movements without software or electronic hardware, but with no opportunity to provide feedback and support; it was also not adapted for people with a motor deficit [24]. By developing T-Chair, a therapy device was created with which patient populations could be treated to help them regain and enhance sitting balance. T-Chair provides feedback and offers increased repetitions with variations, thus allowing for intensive, patient-specific therapy. The T-Chair concept is explained further below.

In this study, we investigated the feasibility, safety, and potential effectiveness of technology-supported sitting balance therapy by using T-Chair. We conducted a single centre pilot randomized controlled trial (RCT) with participants in the chronic phase after stroke with the primary objective of investigating the feasibility and safety of sitting balance therapy enhanced with T-Chair. The secondary objective was to evaluate whether utilizing technology-assisted therapy, in addition to usual care, improved sitting balance, trunk function, mobility, functional balance, strength, and ADL in participants post-stroke, as compared with usual care only.

## Methods

The present study is an assessor-blinded, single centre, parallel-group randomized controlled trial (*ClinicalTrials.gov identifier:* NCT04467554) with ethical approval (Ethische Toestingscommissie Jessa Ziekenhuis, Belgian registration number; B2432020000014). We report this study according to the CONSORT guidelines for a pilot or feasibility trial [25].

### Recruitment, randomization and blinding

Participants were included if the following applied:

1. they had suffered a first stroke more than six months previously;
2. they were 18 years or older;
3. they had impaired trunk function (score ≤ 19 on Trunk Impairment Scale [26]);
4. they were able to maintain a seated position independently for more than 10 s;
5. they were able to travel to the study location;
6. they had no significant comorbidities (other than stroke) affecting trunk function;
7. they had sufficient cognitive and language capacity to understand and perform the study protocol;
8. they provided written informed consent.
9. Participants were excluded if they did not meet one or more inclusion criteria.

Participants were recruited between July and November 2020. Leaflets and posters with study information and contact details were distributed in the rehabilitation centre and in physiotherapy practices near the study location. Written approval was given by the potential participant to be contacted by the investigator (signed informed consent for contact). One investigator contacted potential candidates to further explain the study. After confirming eligibility, written informed consent was obtained from study volunteers.

The study was conducted in a dedicated room in a rehabilitation centre in Belgium, where outpatient therapy is provided. We aimed to recruit 30 participants in the chronic phase after stroke. Because of the pilot nature of the study, a sample size calculation was not required. However, by comparison with previously conducted trials with a similar design, and recommendations by Whitehead et al. [27], a sample of 15 participants in each arm of the trial was considered sufficient to be able to answer our research questions.

The principal investigator (GV) randomly allocated participants, after consent, to two different groups, experimental and control. The principal investigator (GV) used the coin flip randomization method [28] without having any contact with the therapist or participants; allocation was concealed. Information about group allocation was provided to the therapist (EV). Therapist (EV) and participants were aware of the allocated groupings. The assessor and data analyst (LT) was blinded throughout all assessments (three measurement points) and analyses.

### Interventions

Both groups received usual care comprising physiotherapy and/or occupational therapy with strength exercises, conditioning training, and task-oriented therapy. The usual care intensity was between 3 sessions of 30 min and 2 h therapy per week. Therapy was individualized for the needs of each participant by the treating therapists.

#### Control group

Participants in the control group received usual care only, with no time spent on sitting balance therapy.

#### Experimental group

In the experimental group, participants received usual care plus additional technology-supported sitting balance therapy. The experimental therapy consisted of 12 one-hour individual sessions within four weeks at a rate of three to four sessions per week. Each session consisted of 42 min of active sitting balance and trunk training and 8 min of cooling down in a seated position. In the ten remaining minutes, exercises were explained, and questionnaires and feedback recorded. Durations of interventions were monitored by stopwatch and excluded all rest periods and set-up times. Sitting balance therapy was conducted in a seated position and consisted of predefined, standardized exercises, including reaching training, lateral trunk lengthening and shortening, weight-shift training, pelvic tilt exercises, and training while sitting on an unstable surface. The same therapist (EV) trained all participants. The therapist scored safety, of the participant while training, during and at the end of each training session on a 0–10 numerical rating scale (NRS), where higher scores represent better safety. Participants rated tiredness of leg and trunk muscles after each session, also on a 0–10 NRS, where higher scores represent greater fatigue. To determine whether the level of training was too easy, too difficult or just right, safety and tiredness scores were considered after each session. When training was scored as safe (NRS > 5) and tiredness was moderate (an average NRS of < 5), training difficulty was increased to the next level, according to a standardized scheme, evolving to movements with a greater range of motion and/or less stable seated support. Additional file 1 supplies a detailed description of the exercises and cooling down periods of the first session of each week.

### T-Chair

#### T-Chair seating and gaming

**Fig. 1:**
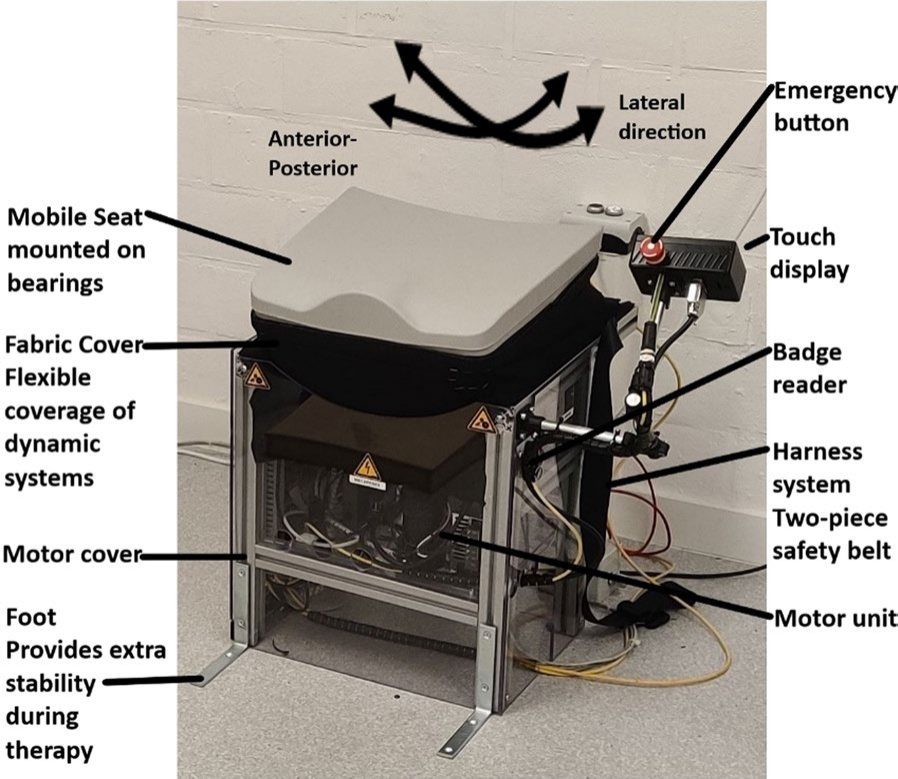
T-Chair prototype and main components

**Fig. 2:**
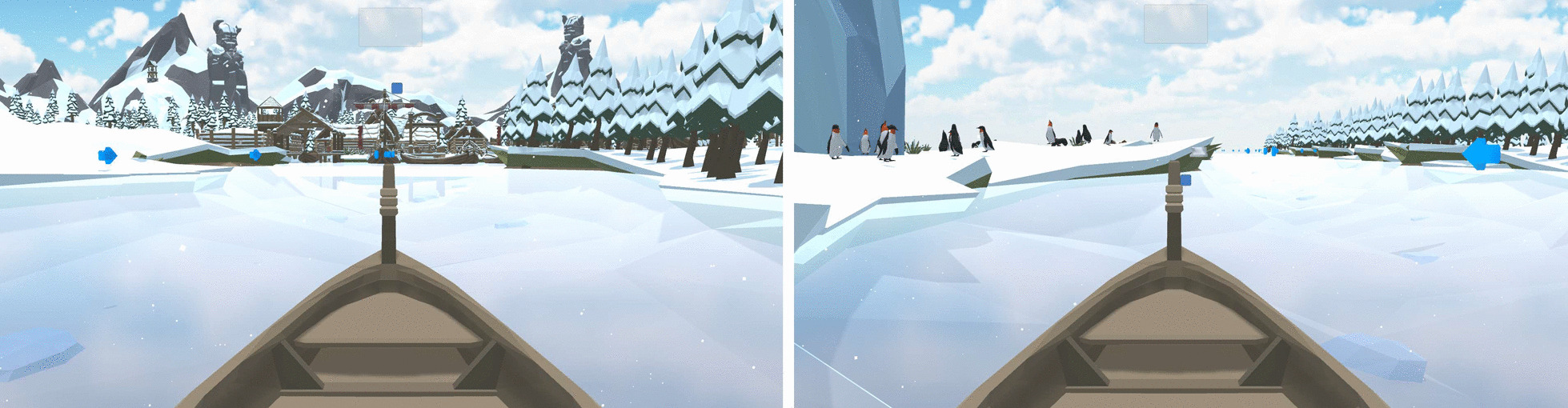
Screenshots of boat game exercise: boat game (left) the participant has to navigate the boat through weight distribution to the left to catch the arrows and then move the boat at the port. Boat game (right), the participant is on the left side of the canal, against the bank, and by weight distribution, the participant can move the boat to the right where a new target is located

Therapy in the experimental group was delivered with a novel rehabilitation technology prototype called T-Chair (Fig. 1). T-Chair is an instrumented robotic chair that provides visual feedback. The seating provides a stable or unstable surface and allows for movements of the seating surface in the anterior–posterior and lateral directions. The seat tilting mechanism consists of two pairs of circular rails, mounted above and perpendicular to each other to allow a relative movement. The first pair of circular rails is mounted in the sagittal plane while the second pair is in the frontal plane. This system allows for a tilting (or spherical movement) of the seat around the frontal and sagittal axis (round arrows on Fig. 1). The construction does not allow the seating to be rotated about the vertical axis. The maximum possible tilt in the anterior–posterior and lateral directions from the starting position is 10°. In the seating, 64 sensors (FlexiForce A401 force sensors, Tekscan, United States) permanently measure the patient's movements when sitting by detecting movements of the centre of pressure. The T-Chair provides visual feedback of range of motion of the centre of pressure during forward, backwards and lateral movements during therapy. The T-chair includes specifically designed gaming to stimulate and activate participants. The goal of the game (boat game, Fig. 2) is to keep balance and improve range of motion of the centre of pressure during weight shifts, according to targets visualized on the screen. The game is developed in ‘Unity’ (Unity Technologies, Denmark) and runs on a UDOO single board computer with Ubuntu (Canonical, United Kingdom**)** as operating system. For the main controlling unit and motion controlling unit, there are also single board computers used. The programs are written in Python. For user feedback, a touch display is used, which is programmed in C. The computer application is written in C# and communicates with an Azure based cloud solution.

For safety, T-Chair is equipped with a safety belt and two emergency stop buttons. The therapist continuously supervised participants during this pilot trial.

#### T-Chair mechanical properties

The device has two casings: a flexible fabric cover that is placed over the dynamic part of the device that allows movement and a stable hard plastic cover that protects the electronics and motor unit. The motor unit consists of the motor unit consists of two hybrid stepper motor units driving the active motion of the T-Chair, together with safety and sensor modules to maintain a safe and well defined control of motion.

A touch display is connected to the device allowing both trainer and participant to control exercises. An internet connection is provided so that training schedules can be saved in the Cloud and software updates can be implemented. The rotational axes of the T-Chair seat is approximately 30 cm above the seat level height. The chair has the following range of motion (ROM) characteristics: forward and backwards movements of 10° (71 mm) each and sideways bending of approximately 10° (73 mm). The seating can be positioned in a horizontal plane or in a stable inclined plane, maximum 10°. The chair's height is 50 cm, its width 55 cm and its depth 90 cm. T-Chair development is based on structured input from participants and clinical experts. A previous study evaluated the usability of this training prototype and provided feedback from therapists and participants (post-stroke patients), leading to further improvements [29].

#### T-Chair features and electronic properties

The training prototype contains emergency stop buttons which can be operated by participant or therapist. All actions are immediately interrupted, making it possible to move the seat manually in all directions, to choose to return to the starting position, or to remove the participant from the seat without further movement of the T-Chair. The power supply remains live during this emergency action. All parts of T-Chair continue to be powered to prevent a new and potentially unsafe situation.

**Fig. 3:**
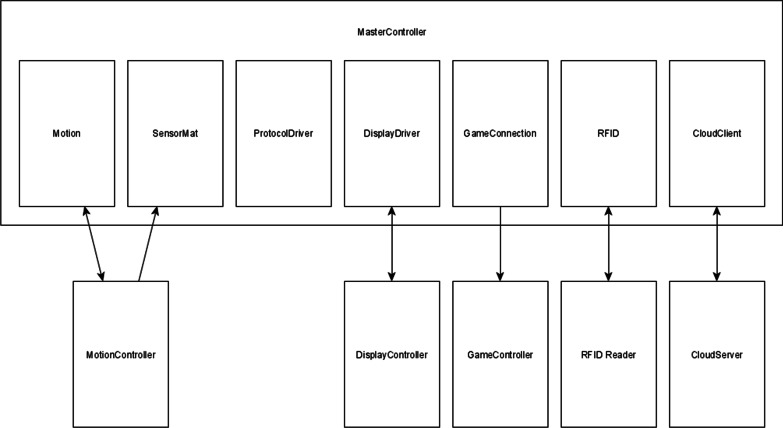
Diagram of the control architecture

A computer application is used together with the chair: its training protocol can be applied to the chair via an RFID badge (USB Desktop reader evohfv2, idtronic, Germany). In the training protocol, the therapist can choose an exercise, adapt its duration, direction and number of repetitions, and synchronise it with the badge. Before starting the therapy, the chair homes in on the starting position: this is followed by placing the badge on the badge reader (NiniX Technologies, Belgium) located on the T-Chair. The T-Chair has five controllers (Fig. 3), each with their own software:

1. the master controller is the main controller of the T-Chair and communicates with the touch display, motion, game, and sensor controllers;
2. the motion controller calculates the centre of pressure and the acceleration, and controls the drives of the motors;
3. the game controller provides all range of motion and sway measurements, and training protocols, as well as affording the occupant the ability to play the boat game;
4. the sensor controller controls all sensors embedded in the seat of the T-Chair. It sends its data to the motion controller for processing;
5. the touch-display controller provides all necessary features for the therapist to interface with the T-Chair.

### Assessments

#### Descriptive baseline characteristics and testing time points

Baseline data, such as age, type and location of stroke, comorbidities, dominant hand, educational level, and gender, were collected. Participants were screened for neglect (star cancellation test [30]) and cognition (Montreal Cognitive Assessment [31]) and level of depression (patient health questionnaire [32]). Testing was performed at three time points for all participants, twice before intervention (termed baseline and pre-intervention), separated by two weeks, and once post-intervention, four weeks after the start of the intervention. The two pre-intervention tests were to determine whether the participants showed stability in the outcomes used in this study. If stability was demonstrated, changes in outcomes in the experimental group after four weeks could be attributed to the study intervention. All outcomes were assessed using clinical measurement tools or questionnaires.

#### Feasibility

The primary aim of this study was to examine the feasibility of the intervention. We evaluated this in terms of recruitment and retention, participation, adherence, acceptability and enjoyment, safety and adverse events, and device development or modification suggestions after each therapy session in the experimental group.

The number of contacted and eligible participants characterized recruitment and retention. We defined recruitment rate as the number of participants in the trial divided by the number of potential participants contacted. We evaluated recruitment rate to see how attractive the idea of the therapy was to potential participants and it gives an indication of how many participants can be recruited from a pool of patients. Retention rate is the number of recruited participants completed all 12 therapy sessions divided by the total number of participants randomized to the experimental group.

The Pittsburgh rehabilitation participation scale [33] assessed participation. The therapist judged participation on a six-point Likert scale, ranging from *poor* to *excellent*. Adherence was evaluated using the Clinician Rating of Compliance Scale [34, 35], a seven-point ordinal scale. A score lower than five is defined as non-adherent; a score of six indicates moderate adherence, with some knowledge and interest, with no prompting required; a score of seven represents active adherence, with the participant showing responsibility for the therapy regimen. Participants scored level of enjoyment during the therapy by means of the physical activity enjoyment scale [36]: this comprises 18 items, each with a seven-point Likert scale, with scores ranging from 18 to 126. The maximum score of 126 represents total enjoyment. Furthermore, all interventions by the therapist to ensure the safety of the participants were recorded after each session. The therapist estimated fatigue using a Visual Analog Fatigue Scale, ranging from zero to ten [37], where zero stood for no fatigue and ten for the worst possible fatigue. General fatigue was enquired about and also specifically fatigue of leg and trunk. The Borg Rating of Perceived Exertion [38] evaluated exertion, its scale ranging from six to 20. A score of six represents no exertion and a score of 20 maximum exertion. Feedback from the participants and the therapist to improve the prototype and protocol were noted after the last therapy session using a questionnaire containing two open and 13 categorical questions, the latter answered with five- or seven-point Likert scales (Additional file 2). This questionnaire asks for instance whether therapy with the prototype has an additional benefit for rehabilitation and whether it was easy to use.

At the end of the last assessment session, all participants completed the Self-Reported Patient Global Impression of Change form [39], which evaluated participants’ belief in improvement by rating their condition as for instance *very much improved*, *not changed* or *very much worse*.

#### Clinical outcome

At all time points, one experienced, blinded assessor (LT) conducted all the assessments.

As sitting balance and trunk training are core components of T-Chair therapy, the primary outcome measure for the clinical data was sitting balance and trunk function. We investigated sitting balance and trunk function using the Trunk Impairment Scale (TIS) [26], evaluating static and dynamic sitting balance and trunk coordination through 17 items on a scale from zero to 23 points. Sitting balance was assessed using the Modified Functional Reaching Test [40]. For this task, a participant sat on a stable surface next to a measuring tape on a wall (leaning against the wall was not allowed). The participant was instructed to reach as far as he/she could with their non-affected hand, without losing stability, in four directions: forwards, to the affected side, to the less affected side, and backwards. The distance reached in each direction was recorded in centimetres.

Gait was assessed in three different areas: gait capacity, speed, and endurance. The Functional Ambulation Categories (FAC) [41] examined walking capacity. This 6-point ordinal scale scores independent walking from level 0 (only able to walk with assistance of at least two therapists) to level 5 (independent walking in- and outdoors, on slopes and stairs). The 10 Metre Walk Test [42] measured comfortable and maximum gait speeds. The Two Minute Walk Test assessed gait endurance. The Fugl-Meyer Assessment of Lower Extremity [43] evaluated selective movements of the lower extremities. The Berg Balance Scale [44] scored functional balance. The Functional Independence Measure [45] and the Modified Barthel Index [46] measured the level of independence in ADL.

We measured trunk and leg strengths (in Newtons) with a hand-held dynamometer (MicroFet 2, Hoggan Health Industries Inc., USA) for trunk extensors, flexors and lateral flexors, hip extensors, flexors, abductors and adductors, knee extensors and flexors and ankle plantar and dorsal flexors. This protocol was based on previous trials [47–49] and adapted to reduce compensation from different muscles.

Tones of different muscle groups were evaluated using the Modified Ashworth Scale [50], including elbow flexors and extensors, hip flexors and adductors, knee flexors and extensors, and ankle plantar flexors. We composed a total score for the affected and non-affected sides.

For all clinical scales, apart from the Modified Ashworth Scale, a higher score represented a better outcome.

All participants received a calendar to note the number of falls and the accompanying circumstances of these, to monitor their usual care, and to record their sporting activities.

### Statistical analysis

The feasibility and safety results are presented descriptively through distributions of response frequencies to the questionnaires and scales.

For changes in clinical outcomes, we evaluated normality of pre-intervention evaluations using the Shapiro–Wilk test and visual inspection of Q-Q plots (because of the modest sample size). Change scores and their variability (pre-intervention minus baseline and post- minus pre-intervention) in both groups were calculated, and we present either mean and standard deviation or median and interquartile range, depending on whether or not data were normally distributed. Differences between the experimental and control groups for baseline and pre-intervention measurements (stability in outcomes before intervention), and for pre-intervention and post-intervention measurements (effect of additional T-Chair therapy) were then analysed using parametric independent t-test or non-parametric independent Mann–Whitney test, depending on normality of distribution. We applied a two-sided p-value < 0.05. Analyses were conducted with IBM SPSS Statistics for Windows, version 27 (IBM Corp., Armonk, N.Y., USA). Analyses were by intention-to-treat and included all randomized participants in the groups to which they were assigned. Dropouts were included if there was a post-intervention assessment, independent of the number of treatments the participant received. This was an exploratory pilot study and hence we did not conduct a multiple testing correction for incorporating multiple outcomes.

## Results

We evaluated feasibility in the experimental group only, through retention, participation, adherence, acceptability and enjoyment, safety and adverse events during training, and device development or suggestions for modification. Clinical outcomes were evaluated in both groups.

### Feasibility and safety

#### Recruitment and retention and baseline characteristics

In total, 41 participants were contacted and assessed for eligibility. Four were excluded and seven decided not to participate. The Covid-19 pandemic situation and travel from home to study location were the main reasons. In total, 30 participants were recruited for this trial (experimental group, n = 15; control group, n = 15). With 30 participants, the recruitment rate was 73%. One participant in the experimental group dropped out (3%): this person had a back injury due to heavy lifting (unrelated to the study) and was unable to continue with the protocol and post-intervention evaluation. The other participants in the experimental group were able to complete all 12 intervention sessions (100%). Retention in the experimental group was high with 14 participants completing all treatment sessions and the final assessment (93%) and 29 completing all evaluations (97%).

**Fig. 4:**
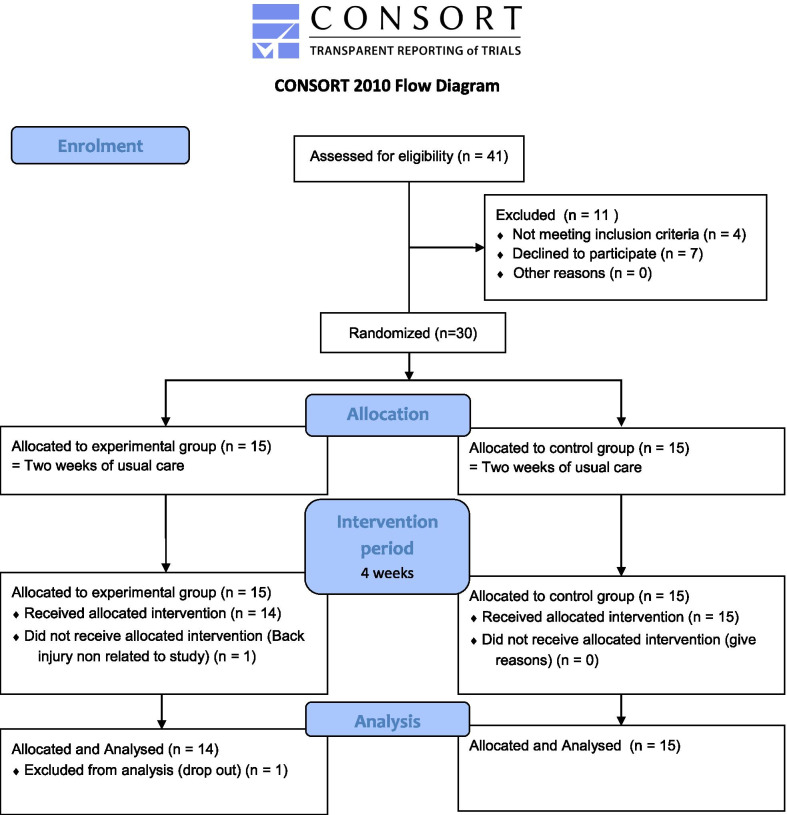
CONSORT flow diagram outlining the distribution of the study participants

Figure 4 presents the flow diagram for the study. Table 1 presents patient characteristics for both groups and shows that there were no significant differences between groups at baseline. There were also no significant differences between groups in hours of usual care received during the four-week intervention period (Table 1, *p* = 0.89).

**Table 1:** Baseline characteristics of both groups

	Experimental group		Control group		
	N = 15		N = 15		p-value independent t-test
Age (mean ± SD)	54.20	11.46	49.07	13.99	0.28
Sex					
Female (n)		7		8	
Male (n)		8		7	
Type of stroke					0.72
Ischaemic (n)		7		10	
Haemorrhagic (n)		7		5	
Hemiplegic side					0.48
Left (n)		8		5	
Right (n)		6		10	
Dominant side					0.33
Left (n)		2		1	
Right (n)		12		13	
Bilateral (n)				1	
Time since stroke in days (mean ± SD)	1913	2834	1177	1375	0.39
phq-9 [0–27] (mean ± SD)	6.47	3.46	3.87	4.31	0.08
Laterality index star cancellation [0–1] (mean ± SD)	0.48	0.07	0.50	0.01	0.33
MoCa [0–30] (mean ± SD)	25.67	4.37	26.33	2.35	0.61
Fall ratio last month (n) (mean + range)	0.29	0–1	0.07	0–1	0.13
2-Minute Walk Test in m (mean ± SD)	104.05	53.54	104.86	48.86	0.97
Functional Ambulation Category					0.33
0 (n)		1		1	
1 (n)		0		0	
2 (n)		0		2	
3 (n)		4		4	
4 (n)		5		8	
5 (n)		4		0	
10 Metre Walk Test, comfortable speed in m/s (mean ± SD)	0.76	0.32	0.81	0.35	0.68
10 Metre Walk Test, maximum speed in m/s (mean ± SD)	1.08	0.49	1.12	0.56	0.82
Trunk Impairment Scale [0–23] (mean ± SD)	11.80	3.10	12.40	3.60	0.63
Fugl-Meyer of Lower Extremities [0–34] (median ± IQR)	24.00	17	24.00	8	0.84
Forward Reach in cm (mean ± SD)	37.42	6.14	41.06	7.8	0.17
Reach to the affected side in cm (mean ± SD)	23.25	7.24	25.58	4.27	0.29
Reach to the less affected side in cm (median ± IQR)	28.25	11.50	28.75	5.00	0.39
Backwards Reach in cm (mean ± SD)	39.18	10.21	39.81	7.11	0.85
Berg Balance Scale [0–56] (median ± IQR)	50	14	50	9	1.00
Functional Independence Measure					
Cognition [5–34] (mean ± SD)	28.27	5.86	31.47	3.83	0.09
Motor [13–91] (median ± IQR)	80	18	81	10	0.87
Total [18–126] (median ± IQR)	107	9	112	21	0.43
Modified Barthel Index [0–20] (median ± IQR)	18	4	19	3	0.49
Total rehabilitation time in hours (mean ± SD)	14.43	15.24	15.23	13.84	0.89

*SD* standard deviation, *IQR* interquartile range, *n* number, *N* total number, *cm* centimeter, *m* metre, *s* seconds, *MoCa* Montreal cognitive assessment, *phq-9* patient health questionnaire

#### Participation and adherence

Participation scores were high during T-Chair training. All participants scored *very good* for their participation during the therapy, with eight participants (53%) rated *excellent* (maximum score). Only one participant was evaluated as *fair to good* during some of the 12 sessions.

Adherence scores were also high, with the median score being 7 out of 7, representing active participation, with participants showing responsibility for the therapy regimen.

#### Enjoyment

On average, participants enjoyed the therapy (range 72–123; maximum possible 126). Five participants had an average score below 100, seven patients scored between 100 and 125, and two patients had average scores higher than 120, for enjoyment.

#### Safety and adverse events

The therapist evaluated safety and recorded adverse events. During and after therapy, a limited number of therapy-related adverse events occurred. One participant fell once during the cooling down period in the first therapy week while not wearing the safety belt, but sustained no injury; three different participants indicated muscle soreness after therapy (shoulder, hip, and back regions).

Fatigue (general, and of the leg and trunk) was found acceptable, given the intensity of the therapy, with mean scores between 5 and 21 (out of 30), corresponding to mild to moderate fatigue. A similar result was noted with the Borg Rating of Perceived Exertion, with mean scores across sessions between 10 and 13.5 (out of 20), indicating that therapy was perceived between fairly light and somewhat hard.

#### Impression of change

In the experimental group, two participants indicated their global perceived condition as *very much improved*, six *much improved*, three *minimally improved* and three *not changed*. In the control group, one participant rated their overall condition as *much improved*, five as *minimally improved*, six as *not changed* and three as *minimally worse*.

#### Participant experience

In the Patient Experience Questionnaire (Additional file 2), all participants in the experimental group indicated that the prototype might bring benefit: all agreed that it was easy to use or were neutral; all agreed that it was enjoyable and felt good to practise with the prototype. Five participants found the explanation of the prototype was sufficient while five thought it could be better. Nine participants thought that therapy with the prototype improved sitting balance, while five were neutral. Three participants would not use the prototype or were neutral, even if it were free of charge, while all the others would use it. The most important aspects of the training, as identified by the participants, were that it was fun to take part, that it focused on improvement and that, throughout the four weeks, the benefits were evident. As limiting factors or improvements, participants mentioned the following: malfunctioning of the prototype hampered the therapy; the seat did not slide properly; seat height should be adjustable; that it was too difficult to use with one hand; that there should be more variation in gaming applications; that feedback could be sent to the participant by e-mail. Overall, each participant found that the intervention met his or her needs.

### Clinical outcomes

**Fig. 5:**
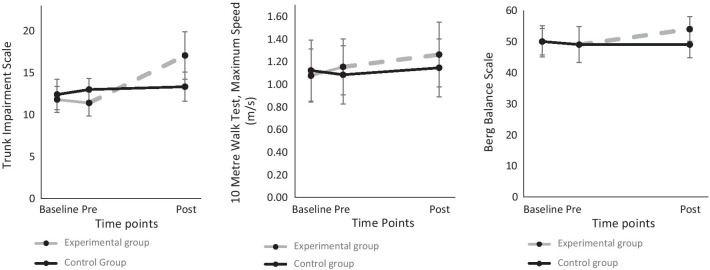
Left: Total TIS score evolution over time (Mean and SD). Middle: Maximum gait speed evolution over time (Mean and SD); Right: Functional balance evolution over time (Median and IQR)

Before the intervention period, no differences between groups were present between the two baseline time points (Additional file 3), apart from walking speed (*p* < 0.004). For trunk function (Trunk Impairment Scale, *p* < 0.001), maximum gait speed (10-Metre Walk Test, *p* < 0.027), and functional balance (Berg Balance Score, *p* < 0.014), significant pre- to post-intervention differences between groups in favour of the experimental group were found (Fig. 5, Table 2, Additional file 4). Overall, improvements were larger in most of the variables in the experimental than in the control group. There were no significant between-group differences in change from pre- to post-intervention for strength and muscle tone outcomes (Additional file 5).

**Table 2:** Between -group analysis on outcomes for trunk function, gait, balance and functional independence

	Pre-intervention			Post-intervention			Change Post- vs. Pre-intervention		
	Experimental group (N = 15)	Control group (N = 15)	*p*	Experimental group (N = 14)	Control group (N = 15)	*p*	Experimental group (N = 14)	Control group (N = 15)	*p*
Trunk Impairment Scale ^A^ [0–23]	11.40 (3.14)	13.00 (2.67)	0.14	18.29 (3.25)	13.33 (3.48)	**0.00**	7.07 (1.69)	0.33 (2.35)	**0.000**
10 Metre Walk Test comfortable speed ^A^ (m/s)	0.87 (0.37)	0.80 (0.35)	0.60	0.89 (0.40)	0.89 (0.36)	0.97	0.06 (0.11)	0.10 (0.07)	0.26
10 Metre Walk Test maximum speed ^A^ (m/s)	1.15 (0.51)	1.08 (0.53)	0.72	1.26 (0.59)	1.15 (0.53)	0.59	0.16 (0.16)	0.06 (0.06)	**0.027**
2 Minute Walk Test ^A^ (m)	106.17 (52.38)	107.41 (49.82)	0.95	113.89 (58.59)	117.59 (54.59)	0.86	11.73 (18.70)	10.18 (12.96)	0.80
Fugl-Meyer of Lower Extremities ^B^ [0–34]	25.00 (14.00)	23.00 (9.00)	1.00	26.00 (11.00)	24.00 (10.00)	0.78	1.00 (3.00)	1.00 (6.00)	0.59
Berg Balance Scale ^B^ [0–56]	49.00 (13)	49.00 (13)	0.81	54.00 (11)	49.00 (9)	0.22	4.5 (5)	0.00 (4)	**0.014**
Functional Independence Measure total score ^B^ [18–126]	107.00 (18)	114.00 (23)	0.37	105.50 (15)	110.00 (21)	0.14	-1.00 (6)	-1.00 (7)	0.78
Modified Barthel Index ^B^ [0–20]	18.00 (4)	19.00 (2)	0.62	18.00 (4)	19.00 (3)	0.65	0.00 (1)	0.00 (1)	0.22

^A^ = mean (Standard deviation), using ANOVA independent *t*-test; ^B^ = median (Interquartile range), using Mann–Whitney *U* test

## Discussion

In this project, we investigated the use of a newly developed therapy device to find out whether it is safe and enjoyable to use, and whether it has a positive effect on motor and functional outcomes, with the potential to increase the intensity of sitting balance training.

Our study demonstrated that technology-supported sitting balance therapy was feasible and safe and, when provided in addition to usual care in the chronic stage after stroke, improved trunk function, gait and functional balance, for community-dwelling stroke survivors.

Improvements in gaming applications could be achieved by including a greater variety of games targeted specifically at patients after stroke. To improve feedback, a standardized report could be generated and sent to the patients via e-mail or an integrated app. Further desirable improvements, such as allowing for one-handed use, making the training more challenging, reducing technical impediments, and providing the resources required for independent training, should be implemented. There were no serious adverse events or other safety issues. The purpose of T-Chair is to enable intensive independent training and it will be more possible to achieve this after incorporation of the feedback that has emerged from our study. In this study, the median score on the Barthel Index for both groups is higher than 18 out of 20, corresponding to a high level of functioning for ADL. Thus this group of patients in the late phase after stroke was able to greatly enjoy this technology-supported sitting balance therapy.

Our results suggest a positive effect for the T-Chair on trunk function, measured with the Trunk Impairment Scale (TIS). Improving trunk function and sitting balance is the primary focus of T-Chair. The experimental group improved a mean 7 points of a maximum 23 (31%) on the TIS, while the control group’s mean improved by only 0.33 points (1.4%). This improvement in favour of the experimental group is clinically relevant. For the TIS, the clinically meaningful difference is 3.5 points in the chronic phase after stroke [51], well below that achieved here and, in fact, all 14 experimental participants surpassed this threshold, compared with only one participant in the control group. This supports our hypothesis that T-Chair, which is specifically designed to train sitting balance and trunk function, achieves its goal.

Our findings also showed that additional sitting balance therapy has a positive effect on maximum gait speed and functional balance. The clinically meaningful difference for gait speed ranges from 0.13 [52] to 0.19 m/s [53]. The mean improvement of 0.16 m/s in the experimental group is therefore in the clinically relevant range. However, our two baseline measures indicate variability in assessment of gait speed and hence we should be careful when interpreting our mean pre- to post-intervention change scores. We also found a significant difference between the two groups for functional balance. The Berg Balance Scale (BBS) addresses many functions not directly targeted with T-Chair therapy, such as balance during 360° turns or alternate placing of foot on a step bench in in standing position. The clinically meaningful difference for the BBS is 12.5 points [54]. In this study, the median change score in the experimental group is about 4.5 points (in the control group zero). This is below the threshold but still a significant difference with the control group. Furthermore, in a systematic review [55] examining the effect of exercise therapy on balance in the chronic stage after stroke, the pooled effect of 28 studies (N = 985) of balance training on BBS showed a mean improvement of 2.2 points (95% CI, 1.26–3.17; *p* < 0.01). Our study demonstrates a median improvement above the upper 95% CI limit, suggesting that additional sitting balance therapy benefits functional standing balance ADL.

Additional therapy was offered to the experimental group, the effect of which on trunk function is similar to previous results. In the trial by An et al. [17] for instance, participants also received additional trunk training in the chronic phase. The intensity in that study was lower, with six hours of therapy in total, compared to 12 h in our study. An et al. not only looked at the effect of training on trunk function, but also on gait and balance, concluding that trunk therapy had a positive effect on trunk, gait, and balance. In other published research, additional trunk therapy has been investigated using technology with feedback. In the most recent study [56], participants in the experimental group received 7.5 h additional canoe-based training with the Wii Sports Resort game (Nintendo®, Kyoto, Japan). The researchers demonstrated a significant post-intervention improvement in reaching towards the affected side, while we found no between-group effect. Studies incorporating technology should focus on the application of rehabilitation-specific techniques, as participants require a dedicated therapy approach and not mainstream gaming which can be challenging for many participants. This finding is confirmed by a systematic review pooling 22 studies [57], where the effects of training with virtual reality technology developed for patients with a stroke (SVR) showed larger effects on body function and activities than did training with nonspecific technology, when compared with control therapy. This is an important value of T-Chair, as it is designed specifically for the large group of patients in need of sitting balance therapy.

Functional performance, measured by the Barthel Index or the Functional Independence Measure, is not commonly used as an outcome measure to evaluate the effect of trunk training. Out of six studies, three [6, 10, 58] found a significant difference between groups and three [20, 59, 60] did not, as in our study. Our finding can be explained by the fact that, at baseline, both groups already had a high level of independence in daily activity (measured with the Barthel Index), while previous studies evaluated the effect on functional performance in the earlier rehabilitation phase, where independence in daily life was more affected. Currently, a Cochrane review and meta-analysis is being performed addressing treatment effects of sitting balance training on functional performance [61]. This will shed light on the possibility of trunk therapy's improving functional independence but this should also be investigated in a future trial.

Based on the results of this study, we calculated a sample size for a further study (with a longer follow-up period and active control intervention) in G*Power 3 [62]. We selected a priori power analyses, the t test family of distributions, and the difference between two independent means as the statistical test. Sample size calculation was based on the evaluated post-intervention score of the outcomes of TIS and its standard deviation for both groups. Based on the effect size of 1.54 found in this pilot study, we would have to include 10 participants in each group to have a power of 90%, with a two-tailed significance level of 0.05. We obtained this sample size in this pilot trial but we would expect the effect size to be smaller when an active control therapy was offered.

Other clinical outcomes, such as strength and reaching, showed only small improvements in the experimental group, and between-group comparisons were not significant. This may be related to the limited sample size, the duration of our therapy protocol, and the fact that our sample was already achieving a high functional level. Nevertheless, when providing therapy, we should consider (functional) goal-specific therapy, based on patient-specific aims. Therapy should focus on training different aspects of these aims. Thus, sitting balance and trunk therapy should be considered as one part of an integrated approach to functional rehabilitation. However, with T-Chair we have a rehabilitation device that allows patients to train independently, thereby reducing the need for continuous therapist supervision and allowing for a more cost-effective approach, with greater rehabilitation intensity. This integrative aspect will be key in future, larger studies.

There are a number of limitations of our study. As in many rehabilitation trials, only the assessor was blinded. The quality of the research might increase if both therapists and participants were blinded. Further, an active intervention in the control group would have the advantage that the limitation of additional versus no additional therapy would be reduced. An alternative would be to conduct a trial where active trunk training in the control group was compared with technology-supported trunk training to determine the effect of the added technology. For this feasibility study, it was decided not to perform a follow-up measurement: as a result, we do not know whether the effects of the therapy were maintained (this should be done in a future study). It is clear from the Barthel Index scores that most participants already had a good to high level of independence in daily life: this limits the generalizability of our findings. Furthermore, the COVID-19 pandemic has affected usual care, with some paused, and other subject to more variability than usual. Nevertheless, we found no between-group differences with respect to hours of usual care. The protocol prescribed conducting the 10 Metre Walk Tests on a sensor mat but, for technical reasons, these data were not usable. For the future, it is important to develop a Phase III study in which intensive training takes place for a longer period, with a larger sample size, with attempts to blind therapists, assessors and participants, and with both groups receiving additional active training.

The strength of this study is the randomized controlled trial design. A homogeneous population, with no significant differences from baseline, was included. It is also positive that there has been a lot of input and user involvement from the target population, which is essential for designing patient-centred care. Recommendations for further development have been formulated as a result of this feasibility study. In addition, the effect of the training on various outcome measures was investigated and it is suggested that additional T-Chair therapy has a positive effect on clinical outcomes, which warrants the further development of technology-supported sitting balance therapy.

## Conclusion

Technology-supported sitting balance therapy, which was specifically developed, based on published research, demonstrated a positive effect on trunk function, gait speed and functional balance in the chronic phase after stroke for people with a high level of independence in ADL. Therapy was feasible and safe, well-accepted by the study population and clinically meaningful. In the future, it would therefore be useful to further develop this technology and therapy programme, so that the final device facilitates a broad range of exercises. There is a need to investigate this device in a large-scale study, where both groups receive additional active therapy.

### Contributions of this literature

- A programme of 12 h of technology-supported sitting balance therapy can improve trunk function, gait speed and functional balance in patients in the chronic phase after stroke.
- Technology developed specifically for a patient population benefits rehabilitation outcomes in people in the late phase after stroke.

## Supplementary Information

**Additional file 1.** Description of the exercises and cooling down of the first session of each week.

**Additional file 2.** Patient experience questionnaire.

**Additional file 3. **Between group analysis on outcome for trunk function, gait, balance and functional independence at baseline and change scores.

**Additional file 4. **Between group analysis on outcome for trunk function, gait, balance and functional independence.

A**dditional file 5. **Between group analysis on outcome on trunk and leg strength and tonus.

## Data Availability

All data generated or analysed during this study are included in this published article.

## Abbreviations

ADL: Activities of daily living
SD: Standard deviation
IQR: Interquartile range
n: Number
N: Total number
NRS: Numerical rating scale
TIS: Trunk Impairment Scale 1.0
CI: Confidence interval
RCT: Randomized controlled trial
phq-9: Patient health questionnaire
MoCa: Montreal cognitive assessment
FAC: Functional ambulation categories
ROM: Range of motion
BBS: Berg balance scale
