# Supplementary material for: Technology-supported sitting balance therapy versus usual care in the chronic stage after stroke: a pilot randomized controlled trial
Source: J Neuroeng Rehabil. 2021 Jul 28;18:120. doi: 10.1186/s12984-021-00910-7 (PMC8316712; doi:10.1186/s12984-021-00910-7)
Supplement: Supplementary file 1 — Additional file 1. Description of the exercises and cooling down of the first session of each week. [file 12984_2021_910_MOESM1_ESM.docx]

*Additional file 1: Description of the exercises and cooling down of the first session of each* *week*

| Week 1 | Week 2 | Week 3 | Week 4 |
| --- | --- | --- | --- |
| Session 1 (both feet stable) | Session 1 (one foot unstable) | Session 1 (two feet on airex) | Session 1 (unstable chair) |
| Calibration measurement sway area, eyes open | Calibration measurement sway area, eyes open | Calibration measurement sway area, eyes open | Tapping blocks (lateroflexion, nearby) |
| Calibration range of motion on stable seat, eyes open | Calibration range of motion on stable seat, eyes open | Calibration range of motion on stable seat, eyes open | Clock yourself |
| Go to target and back to centre on stable seat, 60% of range of motion | Go to target and back to centre on stable seat, 80% of range of motion | Go to target and back to centre on stable seat, 80% of range of motion | Lift and extend leg (unilateral, alternating) |
| Tapping blocks (lateroflexion, nearby), 60% arm reach | Tapping blocks (lateroflexion, nearby), 70% arm reach | Tapping blocks (lateroflexion, nearby), 80% arm reach | Lift Leg and extend leg and homolateral arm |
| Make a clock with arm without moving sway of body | Make a clock with arm without moving sway of body | Make a clock with arm without moving sway of body | Throw tennis balls into bucket (as many as possible) |
| Follow the path unidimensional (5 reps) | Follow the path bidimensional (5 reps) | Follow the path bidimensional (5 reps) | Lift and extend leg (unilateral, alternating) |
| Lift leg (unilateral, alternating) | Lift and extend leg (unilateral, alternating) and contralateral arm | Lift and extend leg (unilateral, alternating) | Lift Leg and extend leg and homolateral arm |
| Lift and extend leg (unilateral, alternating) | Lift Leg and extend leg and homolateral arm | Lift Leg and extend leg and homolateral arm | Trunk rotations lower trunk while lower body is still (4cm) |
| Throw ping bong ball into bucket (as many as possible) | Throw tennis balls into bucket (as many as possible) | Throw tennis balls into bucket (as many as possible) | Trunk rotations upper trunk while upper body is still while extending both arms |
| Boat Game (2x) | Boat Game (2x) | Boat Game (2x) | Look over the shoulders (up) while extending both arms |
| Trunk rotations lower trunk while lower body is still (2cm) | Trunk rotations lower trunk while lower body is still (4cm) | Trunk rotations lower trunk while lower body is still (4cm) | Lift and extend leg (unilateral, alternating) |
| Trunk rotations upper trunk while upper body is still | Trunk rotations upper trunk while upper body is still while extending both arms | Trunk rotations upper trunk while upper body is still while extending both arms | Lift Leg and extend leg and homolateral arm |
| Look over the shoulders (up) | Look over the shoulders (up) while extending both arms | Look over the shoulders (up) while extending both arms | Pattern cones (correct order): place cones on top of each other, 120% arm reach |
| Pattern cones (correct order) | Pattern cones (correct order): place cones on top of each other, 130% arm reach | Pattern cones (correct order): place cones on top of each other, 140% arm reach | Place cones in position, place tennis balls on cones, 120% arm reach |
| Place cones in position, place pingpong balls on cones | Place cones in position, place tennis balls on cones, 130% arm reach | Place cones in position, place tennis balls on cones, 140% arm reach | Recollect tennis balls, recollect cones, 140% arm reach |
| Recollect pingpong balls, recollect cones | Recollect tennis balls, recollect cones, 130% arm reach | Recollect tennis balls, recollect cones, 140% arm reach | Upper trunk rotation while reaching to contralateral side on stable surface, reach to filled bottle (spa) at table height and lift the bottles, 140% arm reach |
| Upper trunk rotation while reaching to contralateral side on stable surface, reach to bottle (spa) at table height | Upper trunk rotation while reaching to contralateral side on stable surface, reach to filled bottle (spa) at table height and lift the bottles, 130% arm reach | Upper trunk rotation while reaching to contralateral side on stable surface, reach to filled bottle (spa) at table height and lift the bottles, 140% arm reach | Resististing the skipping rope (saggital plane) |
| Follow the path unidimensional (5 reps) | Follow the path bidimensional (5reps) | Follow the path bidimensional (5reps) | Pelvic tilting |
| Resisting the skipping rope (sagittal plane) | Resisting the skipping rope (sagittal plane) | Resisting the skipping rope (sagittal plane) | Pelvic tilting (follow sagittal line) while arms go up- and downward |
| Pelvic tilting (follow sagittal line) | Pelvic tilting (follow sagittal line) while arms go up- and downward | Pelvic tilting (follow sagittal line) while arms go up- and downward | Buttock in the seat with mat on the head (follow horizontal line) |
|  | Buttock in the seat with mat on the head (follow horizontal line) | Buttock in the seat with mat on the head (follow horizontal line) | Lateral flexion of the trunk initiated from the shoulder girdle on an unstable surface with hold in end position |
| Follow the path unidimensional (5 reps) | Follow the path bidimensional (5 reps) | Follow the path bidimensional (5 reps) | Balloon in the air (tennis racket) |
| Throwing and recollecting seed bags | Lateral flexion of the trunk initiated from the shoulder girdle on an unstable surface with hold in end position | Lateral flexion of the trunk initiated from the shoulder girdle on an unstable surface with hold in end position | Hula hoop overhead: first head, then thighs and feet |
| Lateral flexion of the trunk initiated from the shoulder girdle on an unstable surface with hold in end position | Follow the path bidimensional (5reps) | Follow the path bidimensional (5reps) | Throwing (in hula hoop) and recollecting seed bags |
|  | Balloon in the air (tennis racket) | Balloon in the air (tennis racket) | Order objects on the shelves, shelves at 140% arm reach; objects left and right alongside the patient (on psoas blocks) |
| Hula hoop overhead: first head, then thighs and feet | Hula hoop overhead: first head, then thighs and feet | Hula hoop overhead: first head, then thighs and feet |  |
| Cooling down | | | |
| 1 minute | [Hands Chest Chair](https://www.tummee.com/yoga-poses/hands-chest-chair): Hands at the height of the chest or supported on the lap. 10 deep belly breathing. | | |
| 1 minute | Neck Bend Exercise: Head up and down 10 times in combination with in and out breaths.  Elevate shoulders 10 times and relax in combination with breathing | | |
| 1 minute | Chair Neck Rolls: Gently move the head from shoulder to shoulder in combination with breathing. | | |
| 1 minute | Neck Twists: Gently turn the head from one side to the other side in combination with breathing. | | |
| 1 minute | [Chair Seated Side Stretch Pose](https://www.tummee.com/yoga-poses/chair-seated-side-stretch-pose): Extend the trunk, hold on one side for 30 seconds, repeat it at both sides. Possibly with arm in extension. | | |
| 1 minute | Chair Seated Twists: turn full trunk to one side and hold for 30 seconds, repeat it at both sides. | | |
| 1 minute | [Chair Pose On Chair Hands On Knees](https://www.tummee.com/yoga-poses/chair-pose-on-chair-hands-on-knees): extend spine upwards, bend slowly forward, stretch the spine while in this forward bend, look up and exhale. | | |
| 1 minute | [Seated Forward Fold Pose on Chair](https://www.tummee.com/yoga-poses/seated-forward-fold-pose-on-chair): exhale, fold forward, chest towards tighs, hands towards floor. | | |
