# Supplementary material for: Technology-supported sitting balance therapy versus usual care in the chronic stage after stroke: a pilot randomized controlled trial
Source: J Neuroeng Rehabil. 2021 Jul 28;18:120. doi: 10.1186/s12984-021-00910-7 (PMC8316712; doi:10.1186/s12984-021-00910-7)
Supplement: Supplementary file 2 — Additional file 2. Patient experience questionnaire. [file 12984_2021_910_MOESM2_ESM.docx]

*Additional file 2 Patient experience questionnaire*

To be completed/ given after the penultimate therapy session.

Overview

**Target**

This 'patient experience questionnaire' was made to map your experience with the use of the T-Chair during one month. The questions were designed to evaluate the convenience, ease of use and enjoyment of using the prototype, in addition to more traditional rehabilitation methods.

Description

This survey takes place after the therapy training. There are 16 questions. This questionnaire takes approximately 15-20 minutes.

**Survey**

Read the questions carefully and put an X in the box corresponding to your answer.

1) The T-Chair brought additional benefit to my post-stroke rehabilitation.

 Totally agree

 Agree

 Neutral

 Disagree

 Disagree at all

2) The T-Chair was easy to use.

 Totally agree

 Agree

 Neutral

 Disagree

 Disagree at all

3) It was a pleasure to work with the T-Chair.

 Totally agree

 Agree

 Neutral

 Disagree

 Disagree at all

4) I felt good about the suggested use of the T-Chair for approximately 60 minutes per therapy session.

 Totally agree

 Agree

 Neutral

 Disagree

 Disagree at all

5) The explanations given during the training were sufficient to use without supervision if necessary.

 Totally agree

 Agree

 Neutral

 Disagree

 Disagree at all

6) I feel like my trunk has gotten stronger using the T-Chair.

 Totally agree

 Agree

 Neutral

 Disagree

 Disagree at all

7) I feel that my trunk is showing improved function during daily life through the use of the T-Chair.

 Totally agree

 Agree

 Neutral

 Disagree

 Disagree at all

8) Which aspect of the T-Chair did you find most useful as an adjunct to standard therapy? Please indicate only 1 answer.

 It was a pleasure to use.

 It focused on the improvement of function during daily activities.

 It can be used at your own pace at home.

 It was adapted to my specific needs.

 The benefits were evident throughout the progress

9) What are the 3 most limiting factors for you when using the T-Chair as an addition to your rehabilitation?

10) What are the 3 most useful aspects of the T-Chair as a supplement to your rehabilitation?

11) If the T-Chair were free of charge, how likely would your use be for further rehabilitation after the study?

 Most likely

 Probably

 Unlikely

 Not likely

 Not at all likely

12) Which of the following terms would you use to describe the T-Chair?

 Reliable

 High quality

 Helpful

 Unique

 Not effective

 Difficult to use

 Unreliable

13) How well did the T-Chair meet your rehabilitation needs, in addition to standard therapy?

 Very good

 Good

 Little good

 Not good

 Not good at all

14) If you could, what changes would you make to the T-Chair?

15) Write your comments or concerns about the T-Chair here.
