# Supplementary material for: Technology-supported sitting balance therapy versus usual care in the chronic stage after stroke: a pilot randomized controlled trial
Source: J Neuroeng Rehabil. 2021 Jul 28;18:120. doi: 10.1186/s12984-021-00910-7 (PMC8316712; doi:10.1186/s12984-021-00910-7)
Supplement: Supplementary file 3 — Additional file 3. Between group analysis on outcome for trunk function, gait, balance and functional independence at baseline and change scores. [file 12984_2021_910_MOESM3_ESM.doc]

Additional file 3 Between group analysis on outcome for trunk function, gait, balance and functional independence at baseline and Change scores

|  | Baseline | | | Change Pre versus baseline | | |
| --- | --- | --- | --- | --- | --- | --- |
|  | Experimental group  (N=15) | Control group  (N=15) | p | Experimental group  (N=15) | Control group  (N=15) | p |
| Trunk Impairment Scale ^A^ [0-23] | 11.80  (3.10) | 12.40  (3.60) | .63 | -.40  (1.60) | .60  (2.23) | .17 |
| 10-Meter Walk Test comfortable speed ^A^ (m/s) | 0.76  (0.32) | 0.81  (0.35) | .68 | 0.11  (0.10) | -0.01  (0.11) | **.004** |
| 10-Meter Walk Test maximum speed ^A^ (m/s) | 1.08  (0.48) | 1.12  (0.56) | .82 | 0.08  (0.12) | -0.04  (0.14) | **.025** |
| 2 Minute Walk Test ^A^ (m) | 104.05  (53.53) | 104.86  (48.86) | .97 | 2.12  (10.37) | 2.55  (11.98) | .92 |
| Forward Reach ^A^ (cm) | 37.42  (6.14) | 41.06  (7.80) | .17 | 2.37  (7.15) | 0.98  (5.36) | .55 |
| Reach to the affected side ^A^ (cm) | 23.25  (7.24) | 25.58  (4.27) | .29 | 0.62  (4.41) | -1.28  (3.00) | .18 |
| Reach to the less affected side ^B^ (cm) | 28.25  (11.50) | 28.75  (5.00) | .39 | 0.50  (5.75) | -2.25  (3.25) | **.015** |
| Backwards Reach ^A^ (cm) | 39.18  (10.21) | 39.82  (7.11) | .85 | -0.20  (7.21) | 0.10  (6.39) | .96 |
| Functional Independence Measure-total score ^B^ [18-126] | 107.00  (9) | 112.00  (21) | .62 | 0.00  (4) | 1.00  (8) | .71 |
| Modified Barthel Index ^B^ [0-20] | 18.00  (4) | 19.00  (3) | .49 | 0.00  (2) | 0.00  (1) | .68 |
| ^A^= mean (Standard deviation) , using independent t-test ^B^ =median (Interquartile range), using Mann-Whitney U test. | | | | | | |
