# Supplementary material for: Technology-supported sitting balance therapy versus usual care in the chronic stage after stroke: a pilot randomized controlled trial
Source: J Neuroeng Rehabil. 2021 Jul 28;18:120. doi: 10.1186/s12984-021-00910-7 (PMC8316712; doi:10.1186/s12984-021-00910-7)
Supplement: Supplementary file 4 — Additional file 4. Between group analysis on outcome for trunk function, gait, balance and functional independence. [file 12984_2021_910_MOESM4_ESM.doc]

Additional file 4 Between group analysis on outcome for trunk function, gait, balance and functional independence.

|  | Baseline | | | Pre intervention | | | Post intervention | | | Change Pre versus. baseline | | | Change Post versus. pre intervention | | |
| --- | --- | --- | --- | --- | --- | --- | --- | --- | --- | --- | --- | --- | --- | --- | --- |
|  | Experimental group  (N=15) | Control group  (N=15) | p | Experimental group  (N=15) | Control group  (N=15) | p | Experimental group  (N=14) | Control group  (N=15) | p | Experimental group  (N=15) | Control group  (N=15) | p | Experimental group  (N=14) | Control group  (N=15) | p |
| Functional Ambulation Category ^B^ (n) | 0 (1)  1 (0)  2 (0)  3 (4)  4 (5)  5 (4) | 0 (1)  1 (0)  2 (2)  3 (4)  4 (8)  5 (0) | .33 | 1 (1)  2 (1)  3 (3)  4 (5)  5 (5) | 1 (1)  2 (0)  3 (2)  4 (4)  5 (8) | .31 | 0 (1)  2 (0)  3 (2)  4 (6)  5 (5) | 1 (1)  2 (0)  3 (2)  4 (4)  5 (8) | .51 | 0🡺1(1)  3🡺2(1) | 0 | 1.00 | 1🡺0(1)  2🡺3(1)  3🡺4 (1)  5🡺4(1)  4🡺5 (1) | 5🡺4 (1)  4🡺5(1) | .38 |
| Forward Reach ^A^ (cm) | 37.42  (6.14) | 41.06  (7.80) | .17 | 39.78  (7.01) | 42.03  (6.13) | .36 | 45.84  (8.74) | 43.77  (7.10) | .49 | 2.37  (7.15) | 0.98  (5.36) | .55 | 6.12  (9.87) | 1.73  (5.95) | 0.16 |
| Reach to the affected side ^A^ (cm) | 23.25  (7.24) | 25.58  (4.27) | .29 | 23.87  (5.31) | 24.30  (4.65) | .81 | 26.68  (4.78) | 27.07  (6.17) | .85 | 0.62  (4.41) | -1.28  (3.00) | .18 | 2.89  (4.62) | 2.77  (7.49) | 0.96 |
| Reach to the less affected side ^B^ (cm) | 28.25  (11.50) | 28.75  (5.00) | .39 | 28.00  (12.25) | 28.25  (8.50) | .49 | 27.88  (13.13) | 25.00  (8.00) | .38 | 0.50  (5.75) | -2.25  (3.25) | **.015** | 0.63  (8.63) | 0.75  (11.00) | .78 |
| Backwards Reach ^A^ (cm) | 39.18  (10.21) | 39.82  (7.11) | .85 | 39.17  (9.57) | 39.92  (10.00) | .84 | 41.39  (9.08) | 38.13  (8.91) | .34 | -0.20  (7.21) | 0.10  (6.39) | .96 | 2.36  (5.29) | -1.78  (7.50) | 0.99 |
| Functional Independence Measure-Motor part ^B^ [13-91] | 80.00  (18) | 81.00  (10) | .87 | 77.00  (13) | 79.00  (18) | .62 | 77.00  (7) | 77.00  (17) | .59 | 0.00  (5) | 1.00  (7) | .54 | -1.00  (6) | 0.00  (5) | .91 |
| Functional Independence Measure-Cognitive part ^A^ [5-35] | 28.27  (5.86) | 31.47  (3.83) | .09 | 30.00  (7) | 33.00  (6) | .13 | 30.00  (8) | 33.00  (4) | .06 | 0.00  (2) | 0.00  (2) | .81 | -0.50  (4) | 0.00  (2) | .51 |

^A^= mean (Standard deviation), using independent t-test ^B^ =median (Interquartile range), using Mann-Whitney U test.
