# Supplementary material for: Technology-supported sitting balance therapy versus usual care in the chronic stage after stroke: a pilot randomized controlled trial
Source: J Neuroeng Rehabil. 2021 Jul 28;18:120. doi: 10.1186/s12984-021-00910-7 (PMC8316712; doi:10.1186/s12984-021-00910-7)
Supplement: Supplementary file 5 — Additional file 5. Between group analysis on outcome on trunk and leg strength and tonus. [file 12984_2021_910_MOESM5_ESM.docx]

Additional file 5 Between group analysis on outcome on trunk and leg strength and tonus

|  | Baseline | | | Pre intervention | | | Post intervention | | | Change Pre versus. baseline | | | Change Post versus. pre intervention | | |
| --- | --- | --- | --- | --- | --- | --- | --- | --- | --- | --- | --- | --- | --- | --- | --- |
|  | Experimental  group  (N=15) | Control  group  (N=15) | p | Experimental  group  (N=15) | Control  group  (N=15) | p | Experimental  group  (N=14) | Control  group  (N=15) | p | Experimental  group  (N=14) | Control  group  (N=15) | p | Experimental  group  (N=15) | Control  group  (N=15) | p |
| Strength trunk extension ^A^  (Newton) | 110.75  (35.96) | 111.92  (21.40) | .91 | 104.64  (27.45) | 119.35  (30.37) | .18 | 129.70  (41.90) | 133.22  (33.51) | .80 | -6.11  (30.27) | 7.43  (26.55) | .20 | 26.11  (26.22) | 13.86  (39.37) | 0.34 |
| Strength trunk flexion ^A^  (Newton) | 95.75  (23.37) | 107.10  (35.84) | .31 | 95.98  (16.17) | 92.83  (15.31) | .49 | 116.46  (25.10) | 106.07  (21.40) | 0.24 | 1.12  (13.82) | -14.27  (27.89) | .07 | 20.46  (17.44) | 13.24  (15.99) | 0.26 |
| Strength trunk lateral flexion affected side ^A^  (Newton) | 111.29  (34.37) | 118.49  (37.73) | .59 | 116.47  (29.84) | 125.18  (39.71) | .72 | 134.71  (26.98) | 135.24  (34.14) | .96 | 9.15  (16.13) | 6.70  (19.66) | .71 | 18.24  (23.80) | 10.05  (31.85) | .44 |
| Strength trunk lateral flexion less affected side ^A^  (Newton) | 156.64  (35.98) | 143.92  (33.81) | .33 | 153.15  (38.40) | 142.05  (26.07) | .34 | 160.65  (17.73) | 146.91  (34.42) | .19 | -3.11  (23.27) | -1.87  (18.00) | .87 | 7.51  (35.24) | 4.86  (29.54) | .83 |
| Strength trunk rotation affected side ^A^  (Newton) | 96.22  (36.43) | 99.30  (33.74) | .81 | 98.13  (29.72) | 94.28  (35.54) | .60 | 115.54  (33.37) | 104.86  (36.15) | .42 | 4.55  (20.94) | -5.02  (20.43) | .22 | 17.41  (38.50) | 10.58  (23.97) | .57 |
| Strength trunk rotation less affected side ^A^  (Newton) | 121.73  (24.72) | 126.41  (32.93) | .66 | 117.52  (23.33) | 119.75  (29.31) | .95 | 121.14  (21.46) | 127.03  (27.99) | .53 | -2.57  (15.19) | -6.66  (23.80) | .58 | 3.61  (23.26) | 7.29  (20.17) | .65 |
| Strength hip extension affected side ^A^  (Newton) | 94.91  (45.74) | 84.77  (22.10) | .45 | 96.88  (37.03) | 92.81  (39.07) | .62 | 119.60  (45.77) | 107.97  (36.85) | .46 | 4.84  (39.15) | 8.04  (32.84) | .81 | 22.71  (36.80) | 15.17  (27.86) | .54 |
| Strength hip flexion affected side ^A^  (Newton) | 114.15  (57.10) | 117.28  (44.84) | .87 | 110.07  (50.06) | 108.49  (45.54) | .87 | 125.05  (50.56) | 113.32  (34.36) | .47 | -2.77  (19.31) | -8.79  (25.05) | .47 | 14.98  (23.82) | 4.83  (24.45) | .27 |
| Strength hip abduction affected side ^A^  (Newton) | 103.16  (36.97) | 117.00  (35.30) | .30 | 99.31  (31.53) | 110.36  (31.79) | .51 | 127.23  (36.43) | 121.13  (26.31) | .61 | -0.71  (24.25) | -6.65  (26.11) | .52 | 27.92  (26.36) | 10.78  (27.30) | .10 |
| Strength hip adduction affected side ^A^  (Newton) | 108.26  (38.28) | 105.40  (38.98) | .84 | 114.89  (40.53) | 102.14  (36.83) | .38 | 126.80  (29.17) | 110.72  (29.20) | .15 | 6.28  (30.77) | -3.26  (25.46) | .36 | 11.91  (29.82) | 8.58  (25.83) | .75 |
| Strength ankle dorsiflexion affected side ^A^  (Newton) | 74.24  (44.60) | 69.77  (37.82) | .77 | 84.25  (44.07) | 73.48  (46.66) | .44 | 91.30  (57.15) | 69.67  (44.08) | .26 | 12.12  (22.28) | 3.71  (26.39) | .35 | 7.05  (26.36) | -3.81  (20.52) | .22 |
| Strength ankle plantar flexion affected side  ^A^  (Newton) | 96.29  (39.67) | 77.26  (50.94) | .26 | 91.13  (36.51) | 87.94  (44.62) | .74 | 115.66  (48.83) | 89.97  (35.68) | .12 | -3.41  (32.18) | 10.67  (27.86) | .21 | 24.54  (36.51) | 2.04  (27.11) | .07 |
| Strength knee extension affected side ^A^  (Newton) | 113.54  (48.23) | 134.63  (53.25) | .27 | 108.47  (44.11) | 121.53  (42.18) | .43 | 127.87  (52.85) | 129.37  (37.29) | .93 | -4.46  (20.62) | -13.09  (36.96) | .44 | 19.40  (30.82) | 7.84  (25.71) | .28 |
| Strength knee flexion affected side ^B^  (Newton) | 78.40  (63.93) | 83.73  (76.23) | .27 | 90.35  (92.82) | 85.67  (84.87) | .84 | 98.80  (115.74) | 79.73  (74.73) | .68 | 16.17  (22.16) | -1.93  (61.50) | .16 | 11.60  (35.07) | 11.57  (26.57) | .68 |
| Strength hip extension less affected side ^B^  (Newton) | 92.37  (57.20) | 99.47  (40.03) | .81 | 109.93  (51.73) | 102.90  (65.70) | .57 | 112.38  (111.61) | 110.27  (77.70) | .81 | -10.97  (28.14) | -26.23  (64.10) | .54 | 8.82  (46.00) | 15.13  (54.57) | .88 |
| Strength hip flexion less affected side ^A^  (Newton) | 165.19  (37.68) | 162.28  (41.86) | .84 | 150.48  (28.37) | 157.59  (33.75) | .50 | 157.47  (33.45) | 150.13  (38.90) | .59 | -15.35  (39.83) | -4.69  (31.19) | .42 | 7.00  (34.24) | -7.46  (30.63) | .24 |
| Strength hip abduction less affected side ^A^  (Newton) | 147.10  (35.39) | 147.08  (44.74) | 1.00 | 139.42  (32.37) | 150.49  (45.99) | .40 | 154.54  (28.26) | 154.40  (55.20) | .99 | -8.82  (28.41) | 3.41  (37.56) | .32 | 15.12  (30.42) | 3.92  (44.09) | .436 |
| Strength hip adduction less affected side ^A^  (Newton) | 152.55  (32.74) | 160.37  (44.24) | .59 | 132.93  (38.02) | 148.52  (37.53) | .30 | 156.08  (26.88) | 151.43  (32.95) | .68 | -18.27  (27.77) | -11.84  (24.91) | .51 | 23.15  (32.98) | 2.90  (33.70) | .11 |
| Strength ankle dorsiflexion less affected side ^A^  (Newton) | 134.90  (24.40) | 134.46  (31.31) | .97 | 131.92  (14.74) | 132.31  (27.87) | .97 | 149.94  (22.89) | 142.18  (32.12) | .46 | -2.25  (26.48) | -2.16 (23.61) | .99 | 18.02  (23.25) | 9.88  (22.76) | .35 |
| Strength ankle plantar flexion less affected side ^A^  (Newton) | 143.71  (31.97) | 138.26  (41.39) | .69 | 139.54  (17.16) | 141.26  (32.05) | .79 | 155.52  (35.31) | 145.50  (43.68) | .51 | -4.94  (31.09) | 3.00  (36.60) | .53 | 15.98  (33.94) | 4.24  (31.77) | .35 |
| Strength knee extension less affected side ^A^  (Newton) | 175.70  (40.80) | 198.95  (74.82) | .30 | 155.28  (35.86) | 166.90  (59.04) | .49 | 171.25  (37.69) | 172.39  (46.20) | .94 | -21.16  (36.53) | -32.04  (42.81) | .46 | 15.97  (42.78) | 5.48  (41.24) | .51 |
| Strength knee flexion less affected side ^B^  (Newton) | 170  (78.13) | 173.63  (64.67) | .94 | 144.32  (59.72) | 146.50  (55.40) | .60 | 152.40  (57.03) | 166.93  (45.53) | .20 | -10.96  (28.14) | -26.23  (64.10) | .37 | 3.05  (42.58) | 16.63  (53.97) | .17 |
| Tone affected side ^B^ | 5.00  (7.00) | 3.70  (7.00) | .39 | 3.00  (5.5) | 5.00  (5.00) | .46 | 3.25  (3.30) | 5.00  (7.50) | .84 | -1.00  (3.50) | 1.00  (2.00) | .009 | .50  (2.10) | 0.00  (4.00) | .91 |
| Tone less affected side ^B^ | 0.00  (1.00) | 0.00  (0.00) | .44 | 0.00  (2.00) | 0.00  (0.00) | .13 | 0.00  (2.00) | 0.00  (0.00) | .62 | 0.00  (0.90) | 0.00  (0.00) | 1.00 | 0.00  (0.60) | 0.00  (0.00) | .40 |

^A^= mean (Standard deviation) , using independent t-test ^B^ =median (Interquartile range), using Mann-Whitney U test.
